# Supplementary material for: The half-life of the bone-derived hormone osteocalcin is regulated through O-glycosylation in mice, but not in humans
Source: eLife. 2020 Dec 7;9:e61174. doi: 10.7554/eLife.61174 (PMC7822592; doi:10.7554/eLife.61174)
Supplement: Reporting standard 1. [file elife-61174-repstand1.docx]

**Check list for the ‘’Reporting guidelines for mass spectrometry’’**

1. General features

1.1 Global descriptors

– Responsible person (or institutional role if more appropriate); provide name, affiliation and stable contact information

– Instrument manufacturer and model

– Customisations (summary): **NA**

2. Ion sources

As each spectrum is acquired using only one ionisation source, select the one that applies

2.1 Electrospray Ionisation (ESI)

– Supply type (static, or fed)

– Interface manufacturer, model

– Sprayer type, manufacturer, model

– Other parameters if discriminant for the experiment **NA**

2.2 MALDI

– Plate composition (or type) **NA**

– Matrix composittion **NA**

– PSD (or LID/ISD) summary, if performed **NA**

– Laser type and wavelength, **NA**

– Other laser and source related parameters, if discriminating for the experiment **NA**

2.3 Other ionisation source

– Description of the ion source and relevant parameters **NA**

3. Post-source component **NA**

As an MS spectrum or chromatogram performed on one instrument cannot be acquired using all existing

analysers and detectors, select the elements that apply

3.1 Analyser

– Ion optics, ‘simple’ quadrupole, hexapole, Paul trap, linear trap, magnetic sector, FTICR, Orbitrap: name of the analyser(s)

– Time-of-flight drift tube (TOF): Reflectron status **NA**

3.2 Activation / dissociation

The associated acquisition parameters are covered in 4.1

– Instrument component where the activation / dissociation occurs

– Gas type (when used)

– Activation / dissociation type

4. Spectrum and peak list generation and annotation

4.1 Data acquisition

– Software name and version

– Acquisition parameters

4.2 Data analysis

– Software name and version

– Parameters used in the generation of peak lists or processed spectra **NA**

4.3 Resulting data

The following information should be provided for each dataset

– Location of source (‚raw‛) and processed files

– The chromatogram(s) for SRM data and other relevant cases **NA**

The following information should be provided for each spectrum or peaklist

– m/z and intensity values

– MS level

– Ion mode

– For MS level 2 and higher, precursor m/z and charge if known, with the full mass spectrum / peaklist containing that precursor peak, where available
